# Global perspective of Innovation in Hip and Knee Replacement in 2020

**DOI:** 10.1051/sicotj/2020043

**Published:** 2020-12-15

**Authors:** Cécile Batailler, Jacques Caton, Sébastien Lustig

**Affiliations:** 1 FIFA Medical Center of Excellence, Orthopaedic Surgery and Sports Medicine Department, Croix Rousse Hospital, Civil Hospices of Lyon 69004 Lyon France; 2 Groupe Lépine 175 rue Jacquard – CS 50307 69727 Genay Cedex France; 3 LBMC UMR T 9406, Laboratory of Chock Mechanics and Biomechanics, Claude Bernard Lyon 1 University 69003 Lyon France

The knowledge of new technologies and current concepts are primordial in orthopaedic surgery. That is necessary to progress and improve our current practice continuously. This special issue of SICOT-J about hip and knee arthroplasty consists of 12 original articles on orthopaedic advances or controversial subjects, such as robotic-assisted surgeries, alignment of total knee arthroplasty (TKA), cementless tibial implants, polyethylene or ceramic in total hip arthroplasty, return to sport and arthroplasty…

Kouyoumdjian et al. reported the current concepts in robotic-assisted total hip arthroplasty. The advantages and the impact of robotic-assisted surgery in knee arthroplasty has been often described in recent literature. Nevertheless, the impact of robotic-assisted system for hip arthroplasty is less known. This paper brings a clear state of knowledge about this new technology [1].

The materials used in total hip arthroplasty remain a relevant issue. In a comparative study on highly cross-linked polyethylene (XLPE) and ultra-high molecular weight polyethylene (UHMWPE), Carli et al. showed that annual linear wear rates were significantly higher with UHMWPE (0.21 mm/year) compared to XLPE (0.05 mm/year) in a simple mobility in total hip arthroplasty [2]. XLPE coupled with oxidized zirconium femoral heads had minimal wear and no osteolysis at 10-year follow-up. In a relevant update, Langlois and Hamadouche reported the major and recent knowledges on crosslinked polyethylene in total hip arthroplasty [3]. While Bauwens et al. reported the earlier results of ceramic-on-ceramic bearing in total hip arthroplasty by direct anterior approach [4]. Neri et al., described with a pragmatic way the tips and tricks of the dual mobility in total hip arthroplasty [5]. This article brings interesting and relevant advices and concepts to improve the right use of dual mobility.

The surgical approach in total hip arthroplasty can also evolve, but this evolution can become difficult according to the complexity of surgical techniques. Foissey et al. have described the learning curve of the anterior approach in total hip arthroplasty according to the surgeon experience [6].

Five published articles reported interesting results about knee arthroplasty. Pacoret et al. described the survival rate of the tibial component in cementless TKA at a mean follow-up of 8 years, with a minimal follow-up of 5 years [7]. Indeed, it is now accepted that cementless implantation of the femoral component provides equivalent results to cemented one, however, the optimal fixation method of the tibial component remains controversial. This comparative study reports a very satisfying survival rate in both groups cemented and cementless TKA.

In addition to implants fixation, two articles introduce the impact of alignment in TKA. In a systematic review, Sappey-Marinier et al. assessed five prospective randomized controlled trials comparing clinical and radiological outcomes, and complications in TKA with kinematic alignment and mechanical alignment [8]. Kinematic alignment in TKA achieved clinical and radiological results similar to those of mechanical alignment. The complication rate was not increased. Assi et al. were warning that the intra-medullary guide can yield sub-optimal corrections, particularly for severe tibial varus in TKA [9].

The osteotomies around the knee can impact the surgical strategy of TKA and their outcomes. In a relevant systematic review, Luceri et al. reported the results of five useful studies on TKA after distal femoral osteotomy [10]. They concluded on the complexity of this surgery and the need of specific technical tips and tricks to obtain an accurate knee balancing. But future prospective studies and comparative trials are yet necessary to address the impact of distal femoral osteotomy in knee replacement surgery.

In the last study, Plassard et al. described the return to sport after TKA and assessed its predictive factors in a large cohort of TKA [11]. Preoperative condition and activity are the two most significant predictive factors for return to sport.

The current concepts and innovations can also settle in the treatment of prosthetic-joint infection. In a didactic article, Ferry et al. described the medical innovations to maintain the function in patients with chronic prosthetic-joint infection for whom explantation is not desirable: phage therapy and phage lysins [12].

There is no doubt that these technologies and current concepts are here to stay and as they improve, they will change the operating theatre of the future and the outcomes for our patients. We hope that you will enjoy reading this special issue of SICOT-J about hip and knee arthroplasty.

Good reading.
